# Genome sequence analysis of *emm*89 *Streptococcus pyogenes* strains causing infections in Scotland, 2010–2016

**DOI:** 10.1099/jmm.0.000622

**Published:** 2017-11-03

**Authors:** Stephen B. Beres, Randall J. Olsen, Matthew Ojeda Saavedra, Roisin Ure, Arlene Reynolds, Diane S. J. Lindsay, Andrew J. Smith, James M. Musser

**Affiliations:** ^1^​ Center for Molecular and Translational Human Infectious Diseases Research, Department of Pathology and Genomic Medicine, Houston Methodist Research Institute, and Houston Methodist Hospital, Houston, TX 77030, USA; ^2^​ Departments of Pathology and Laboratory Medicine and Microbiology and Immunology, Weill Cornell Medical College, NY 10021, USA; ^3^​ Scottish Haemophilus Legionella Meningococcus Pneumococcus Reference Laboratory, New Lister Building, Glasgow, G31 2ER, Scotland, UK; ^4^​ College of Medical, Veterinary and Life Sciences, Glasgow Dental Hospital and School, University of Glasgow, 378 Sauchiehall Street, Glasgow, G2 3JZ, Scotland, UK

**Keywords:** population genomics, microbial epidemiology, population, *Streptococcus pyogenes*

## Abstract

**Purpose:**

Strains of type *emm*89 *Streptococcus pyogenes* have recently increased in frequency as a cause of human infections in several countries in Europe and North America. This increase has been molecular epidemiologically linked with the emergence of a new genetically distinct clone, designated clade 3. We sought to extend our understanding of this epidemic behavior by the genetic characterization of type *emm*89 strains responsible in recent years for an increased frequency of infections in Scotland.

**Methodology:**

We sequenced the genomes of a retrospective cohort of 122 *emm*89 strains recovered from patients with invasive and noninvasive infections throughout Scotland during 2010 to 2016.

**Results:**

All but one of the 122 *emm*89 infection isolates are of the recently emerged epidemic clade 3 clonal lineage. The Scotland isolates are closely related to and not genetically distinct from recent *emm*89 strains from England, they constitute a single genetic population.

**Conclusions:**

The clade 3 clone causes virtually all-contemporary *emm*89 infections in Scotland. These findings add Scotland to a growing list of countries of Europe and North America where, by whole genome sequencing, *emm*89 clade 3 strains have been demonstrated to be the cause of an ongoing epidemic of invasive infections and to be genetically related due to descent from a recent common progenitor.

## Introduction

*Streptococcus pyogenes*, a strict human pathogen, causes highly diverse infections that range in severity from common self-limiting skin and throat infections (impetigo and pharyngitis), to less common invasive infections that have high morbidity and mortality such as scarlet fever, sepsis and necrotizing fasciitis [1]. For decades, *S. pyogenes* strains were subtyped based on serologic diversity in the M protein, a highly polymorphic surface-displayed molecule that is an important virulence factor involved in multiple host–pathogen interactions, including adherence and resistance to phagocytosis [2]. Over the last 20 years, a shift away from M protein serotyping to *emm* gene sequence typing has occurred. Greater than 220 distinct *emm* types have been described based on allelic variation in the region of the *emm* gene that encodes the highly polymorphic amino terminal 50 amino acids of the mature M protein [3].

Considerable new information has been obtained by *emm* gene sequence typing. However, whole genome sequence analysis of approximately 10 000 *S. pyogenes* strains [4–15] has revealed that polymorphisms exist among strains classified by *emm* type and found that the genome of nearly every strain is genetically distinct. The largest contributors to this genome-to-genome variation are mobile genetic elements (MGE), such as prophages and integrative conjugative elements (ICE), and small sequence differences such as single nucleotide polymorphisms (SNPs) and short insertions and deletions (indels). The genome sequence data provide a rich source of information for epidemiologic and pathogenesis studies framed within an evolutionary population genomic context.

In recent years, strains of *emm*89 *S. pyogenes* have been reported as a cause of increasing disease frequency in many countries worldwide, and have been documented to be a cause of local disease outbreaks [6, 15–43]. Genome sequence analysis of almost 1500 *emm*89 strains from the USA, Finland, Iceland, Canada (Ontario), the UK (the great majority from England) and Portugal has identified three major genetically distinct strain clusters, designated clades 1, 2 and 3 [6, 14, 15, 19, 24, 41]. Clade 1 strains have been identified only in the USA and Canada. Importantly, concurrent with a significant increase in the number and frequency of invasive *emm*89 infections, clade 3 strains emerged in the early 2000s and around 2007–2009 expanded rapidly in the USA, Finland and Iceland, displacing their predecessor clade 1 and 2 strains in these countries [6, 41]. Similarly, clade 3 organisms have become abundant in England, where they also displaced clade 2 organisms [15]. The *emm*89 clades differ predominantly due to multiple horizontal genetic transfer (HGT) events, several of which involve known virulence factors [6, 15]. Moreover, whole genome RNAseq expression analysis found that these regions of recombination were significantly non-randomly associated with altered gene expression [6].

The goal of the present study was to analyse the genome sequences of all available *emm*89 strains causing infections in Scotland between 2010 and 2016. We also sought to determine if geographic and temporal variation occurred in the genomic types of organisms causing disease in the country. Here we report that with a single exception, recently emergent clade 3 strains caused all invasive *emm*89 infections in Scotland during the study period. There is restricted evidence of genetic substructure of *emm*89 clade 3 subclones within Scotland and other regions of the UK.

## Methods

### Bacterial strains

The 122 *emm*89 strains newly sequenced and studied herein were cultured from individuals throughout Scotland between 2010 and 2016, including 98 from patients with symptomatic infections at normally sterile sites (designated invasive infection isolates). Strains were identified as *emm*89 by *emm* gene sequencing as described [44], and confirmed to be *emm*89 based on whole genome sequence data (see below). The strain sample includes virtually all *emm*89 organisms causing invasive infections in Scotland from 2013 onward. The strain samples from 2010 through 2012 were largely from Glasgow and Lanarkshire, that is, the west central belt of Scotland (Fig. S1, available in the online version of this article). The sample also included 24 strains cultured from throats of individuals with pharyngitis or asymptomatic colonization (designated non-invasive infection isolates). The strain, year, infection type, geographic location and phage-profile are presented in Table S1.

For the purpose of epidemiologic and genetic comparisons, genome data from 133 previously sequenced *emm*89 *S. pyogenes* strains [6, 15, 41] were also used in this investigation, and are referenced at the appropriate places below.

### Multiplexed genome sequencing

Bacterial cells were lysed with mutanolysin and lysozyme (Sigma, St. Louis, MO) and chromosomal DNA was extracted with the DNeasy 96 Blood and Tissue Kit (Qiagen, Germantown, MD). The extracted DNA was quantified with a Qubit 3 Fluorometer using a Qubit dsDNA BR Assay Kit (Invitrogen, Carlsbad, CA). Sequencing libraries were generated by tagmentation from the extracted DNA with the Nextera XT DNA Sample Preparation Kit and Index Kit V2 (Illumina, San Diego, CA). The DNA concentration of the resultant individual index tagged libraries was measured with a Qubit dsDNA HS Assay Kit. The DNA size distribution (quality) of a random subset of the libraries (approximately 5 %), was assessed with a Bioanalyzer instrument using a HS DNA Kit (Agilent, Santa Clara, CA). Libraries in sets of 96 samples were pooled at equivalent concentrations, and paired-end 150 nt sequence reads were obtained from the pooled libraries using a NextSeq 500/550 High Output Reagent v2 300 Cycle Cartridge and an Illumina NextSeq instrument. Data for the 122 newly sequenced *emm*89 genomes were deposited in the NCBI Sequence Read Archive under accession number PRNJNA387243.

### Data preprocessing and initial genetic characterization

Before the sequence data were used in subsequent genetic analyses, reads were preprocessed to remove low quality sequences and library generation artifacts (e.g. adaptor contamination) with Trimmomatic [45], and base call errors were corrected with Musket [46]. Reads were then assembled *de novo* with SPAdes [47]. The *S. pyogenes* genome is approximately 2 Mbp in length. Strains for which the *de novo* assembly was larger than 2.2 Mbp were considered potentially contaminated and were reisolated and resequenced (*n*=9, 5 from the throat a non-sterile site of isolation) or excluded (*n*=2, viable pure cultures were not obtained) from further analysis. The resultant high-quality and contaminant-free reads were used in subsequent genetic analyses. The multi-locus sequence type (https://pubmlst.org/spyogenes/), *emm* type (ftp://ftp.cdc.gov/pub/infectious_diseases/biotech/tsemm/), presence of hyaluronate capsule synthesis genes (*hasABC*) and presence of potential antibiotic resistance genes (http://en.mediterranee-infection.com/article.php?laref=283%26titre=arg-annot) were assessed using SRST2 [48] with the indicated databases. This initial genetic typing confirmed the species and *emm* type of the strains studied.

### Polymorphism discovery

Preprocessed reads were aligned to the genome of *emm*89 clade 2 reference strain MGAS23530 (GenBank accession number CP013839) using SMALT (http://sourceforge.net/projects/smalt/). Sequence differences between the aligned reads and the reference genome were identified with FreeBayes [49]. These polymorphism data were filtered on the basis of alternate base call consensus (≥70 %), mapped quality (≥Q30) and coverage depth (≥10-fold) using VCFfilter (https://github.com/ekg/vcflib#vcflib). Since the genome of strain MGAS23530 lacks prophages and integrative and conjugative elements, the resultant polymorphism calls were made relative to only the core chromosome, thereby obviating the need to exclude phylogenetic inference potentially distorting polymorphisms within MGEs.

### Phylogenetic inference and population structure

SNPs (i.e. variant site nucleotides) identified relative to the 1709394 genome of *emm*89 clade 2 reference strain MGAS23530 along with all invariant site nucleotides were concatenated in sequential order to generate entire core genome aligned sequences for each strain studied using Prephix, Phrecon and SNPswapper (https://github.com/codinghedgehog). Based on the 1709394 nucleotide full-length core genome aligned sequences, regions of putative horizontal transfer were predicted and polymorphisms within these regions were removed using Gubbins [50]. To constrain the inferences to primarily vertically inherited nucleotide differences, all phylogenetic reconstructions presented are based on Gubbins-filtered core genome SNPs. Phylogeny among strains was inferred by the neighbour-joining method with SplitsTree [51], and tree figures were generated with Dendroscope [52]. Genetic distances between strains (pairwise and overall mean SNP differences) were determined with mega [53]. Root-to-tip genetic distances were determined using TempEst [54].

### Gene content and MGE analysis

Total gene and MGE content were assessed for the strains studied relative to the non-redundant pangenome gene content defined for 30 complete *S. pyogenes* genomes representing 18 *emm* types, as previously described, with minor modifications [6]. Briefly, reads were mapped to the pangenome (denoted GAS-30) with SMALT and the mean depth of coverage was determined with BEDTools2 [55] for all 2835 gene clusters represented. To compensate for differences in the depth of sequencing, depth-of-coverage for reads mapped relative to genome of reference strain MGAS23530 was normalized to 100-fold. Based on mapping of the sequencing reads from the clade 3 reference genome MGAS27061 to the GAS-30 pangenome, a normalized mean DOC value of >10 corresponded with gene presence and <10 with gene absence. The resultant gene content data were used to infer phage presence or absence, which was expressed as prophage content profiles or prophage genotypes as previously defined for 1193 *emm*89 strains [6]. A prophage was called present if a minimum of 75 percent of its gene content represented in the GAS-30 pangenome was determined to be present. Reads not mapping to the GAS-30 pangenome were assembled *de novo* with SPAdes. Resultant contigs greater than 100 nucleotides were queried against the NCBI non-redundant database using blast to determine their nature.

## Results

### Genome sequencing and initial analysis

**Fig. 1.**
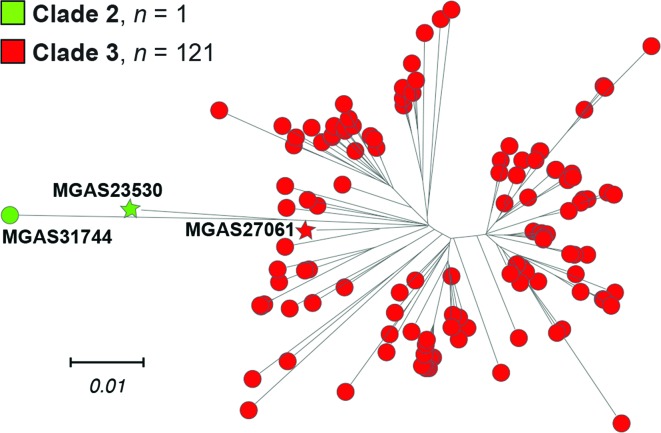
Genetic relationships among the Scotland *emm*89 strains. The reconstructed phylogeny for 122 Scotland *emm*89 strains in conjunction with *emm*89 clade 2 and clade 3 reference strains, MGAS23530 and MGAS27061 respectively, inferred by the neighbour-joining method based on 1410 core chromosomal SNPs, is shown. To constrain the inference to primarily vertically inherited SNPs, SNPs were filtered with Gubbins to exclude sites predicted to be horizontally acquired. The tree is rooted on clade 2 reference strain MGAS23530. The mean genetic distance in nucleotide differences (i.e. SNPs strain-to-strain) among the 121 clade 3 strains is 48.1, and between clade 2 strain MGAS31744 and the clade 3 strains as a group is 107.7.

The genomes of all 122 strains from individuals from Scotland (*n*=98 invasive disease isolates and *n*=24 throat non-invasive isolates) were sequenced to a mean 231-fold depth-of-coverage (range, 108-fold–519-fold) using a paired-end 150 nt read strategy, and polymorphisms were identified. Inference of genetic relationships using core chromosomal SNPs found that these *emm*89 strains from Scotland have a major population of 121 strains, and a single genetically divergent outlier strain (Fig. 1). Phylogenetic analysis showed the major *emm*89 population of 121 strains corresponded to previously identified epidemic clade 3, whereas the single outlier strain (MGAS31744) was a preepidemic clade 2 organism (Fig. 1).

### Variation in gene content and prophage genotypes

**Fig. 2.**
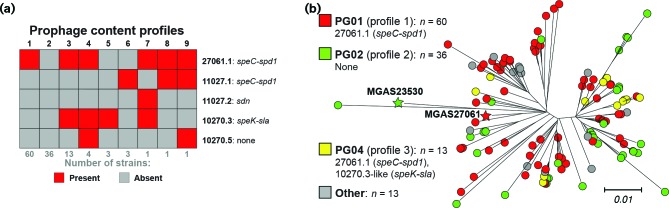
Prophage content of the Scotland *emm*89 strains. (a) A matrix of the five prophages and the nine different profiles of prophage content identified among the Scotland isolates is illustrated. The three most prevalent profiles 1, 2 and 3 correspond respectively with prophage genotypes PG01, PG02 and PG04, previously defined for 1193 *emm*89 strains [6]. (b) The phylogeny of the 122 Scotland strains inferred by neighbour-joining based on 1410 core SNPs is shown. The tree is rooted on clade 2 reference strain MGAS23530. Strains are coloured by their prohage genotype as indicated in the index. Differences in the tree among closely genetically related strains are evident in prophage content, consistent with prophage gain and loss events.

**Fig. 3.**
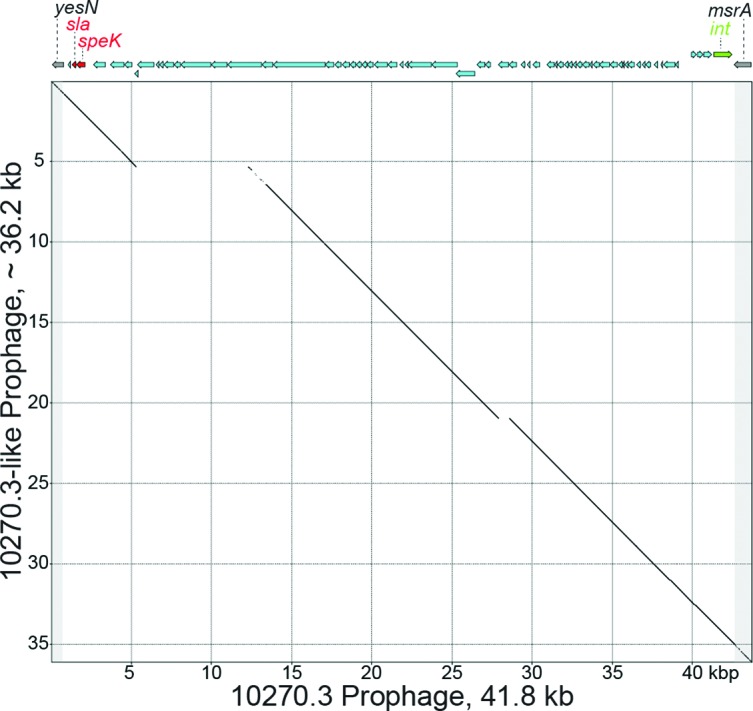
SpeK and Sla encoding prophages. A dot matrix plot comparison of SpeK-Sla encoding prophages from Scotland *emm*89 strain MGAS31644 and *emm*2 reference strain MGAS10270 (GenBank accession number CP000260.1) is shown. The *emm*89 10270.3-like prophage was partially assembled *de novo* on two contigs using SPAdes. The diagonal line indicates portions of the prophages that share 99 % or greater sequence identity over a 50 nucleotide moving window. High sequence identity is shared over nearly the full-length of the prophages compared.

We, and others, have shown that *emm*89 *S. pyogenes* strains can vary in gene content as a consequence of HGT and differences in MGE content [6, 15, 41]. The gene content (i.e. gene presence or absence) of the 122 Scotland *emm*89 strains was determined relative to the pangenome constructed from the combined gene content (>53 000 genes) of 30 *S. pyogenes* genomes of 18 *emm* types [6]. No ICEs were detected in the 122 strains. Virtually all differences in gene content are due to strain-to-strain differences in the five prophages detected among the 122 strains (Fig. 2a). Our analysis identified nine different prophage content profiles or prophage genotypes (Fig. 2a). The three more prevalent prophage genotypes are each present in 13 or more strains and account for 109 (89.3 %) of all 122 isolates (Fig. 2b). The remaining six prophage genotypes are each present in four or fewer strains and account for only 13 (10.7 %) of the isolates. The three most prevalent prophage genotypes (PG01, PG02 and PG04) among these Scotland isolates are those previously identified to be prevalent among 756 *emm*89 clade 3 strains in the USA and Finland [6]. The most prevalent prophage genotype (PG01) was found in 60 (49.1 %) of the 122 isolates. Clade 3 reference strain MGAS27061 has prophage genotype PG01. PG01 is characterized by the presence of one prophage (ϕ27061.1) encoding two virulence factors, superantigen pyrogenic exotoxin SpeC and secreted DNase Spd1. The second most prevalent prophage genotype (PG02), was found in 36 (29.5 %) isolates, and is characterized by a lack of prophages in the genome. Clade 2 reference strain MGAS23530 (GenBank accession number CP013839) has prophage genotype PG02. The third most prevalent type (PG04), was found in 13 (10.7 %) isolates, and consists of prophage 27061.1 and a second prophage integrated into the genome at the two-component system regulator *yesMN*. This prophage shares substantial gene content with prophage 10270.3, and both encode the virulence factors' superantigen pyrogenic exotoxin SpeK and secreted phospholipase Sla (Fig. 3). The intermingling of these three prophage genotypes on multiple branches of the tree is consistent with these prophages being dynamically gained and lost in the population (that is, the prophage genotypes are not uniquely associated with a given genetic background). Additionally, as expected, all 121 of the Scotland clade 3 strains lacked the hyaluronic acid capsule synthesis genes (*hasABC*) consistent with previous findings [6, 15, 41, 42].

### Genome analysis of strains cultured from throats

**Fig. 4.**
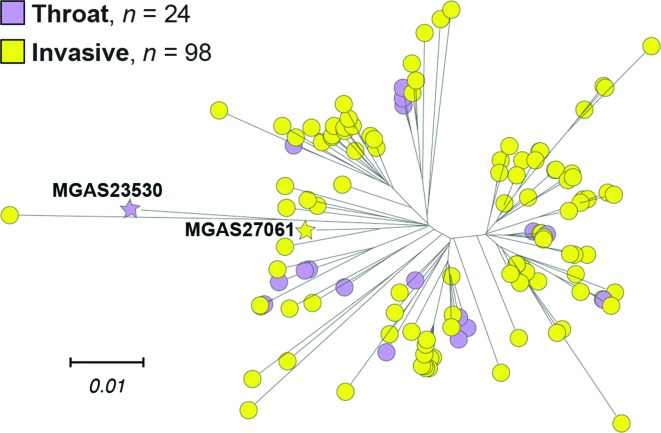
Genetic relationships among Scotland throat and invasive isolates. Reconstructed phylogeny of the 122 Scotland strains inferred by neighbour-joining based on 1410 core SNPs is shown. The tree is rooted on clade 2 reference strain MGAS23530. Strains are coloured by infection, as indicated in the index. Throat and invasive isolates are intermingled throughout the tree, as groups they are not separate and genetically distinct.

Twenty-four organisms cultured from the throat were studied. We found that the throat isolates were genetically intermixed with the invasive isolates. That is, the throat and invasive strains were not separate genetically distinct groups (Fig. 4).

### Genetic structure with respect to year of isolation

**Fig. 5.**
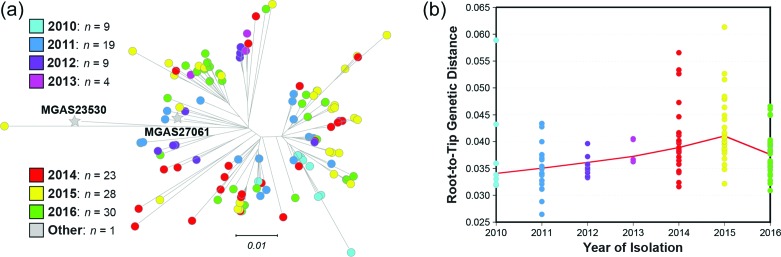
Temporal distribution of the Scotland *emm*89 strains. (a) Reconstructed phylogeny of the 122 Scotland strains inferred by neighbour-joining based on 1410 core SNPs is shown. Strains are coloured by the year of isolation as indicated in the index. In general, strains isolated in earlier years are more proximal and in later years are more distal on the tree branches. (b) A scatter plot of root-to-tip genetic distances for the adjacent 122 Scotland strain tree is shown. The weighted fit shows an overall upward trend and a consistent yearly increase in the root-to-tip distances over 2010–2015.

We examined if a distinct temporal relationship existed between the year of strain isolation and the topology of the phylogenetic tree. In general, isolates from the early years of the Scotland sample tend to be located closer to the root of the tree, whereas those recovered in the later years (2015 and 2016) were located farther toward the tips of the tree branches (Fig. 5a). This impression is supported by analysis of root-to-tip genetic distances for the best-fit rooted tree, which found an overall trend for increasing genetic distance with increasing time since a point in the past (Fig. 5b).

### Comparison of the genetic structure of strains from Scotland and other regions of the UK

**Fig. 6.**
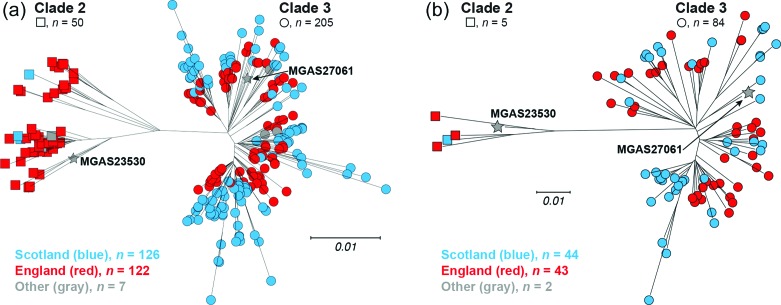
Genetic relationships among Scotland and England *emm*89 strains. (a) Reconstructed phylogeny of 255 *emm*89 strains inferred by neighbour-joining based on 2766 Gubbins-filtered core SNPs is shown. Strains are coloured by country, Scotland in blue and England in red. Consistent with the earlier period of collection of the England strain cohort (*ca.* 2003–2013) relative to the Scotland strain cohort (*ca.* 2010–2016), one-third (41/122) of the England strains are of preepidemic clade 2, whereas only 3 of the 126 Scotland strains are clade 2. Moreover, in the epidemic clade 3 portion of the tree (i.e. the right side), by in large the England strains are more proximal on the branches than the Scotland strains. (b) Reconstructed phylogeny of 89 *emm*89 strains inferred by neighbour-joining based on 1087 Gubbins-filtered core SNPs is shown. The 44 Scotland and 43 England strains are temporally matched sets of all strains collected between 2010–2013.

We next tested the hypothesis that *emm*89 strains recovered in Scotland were a distinct genetic subpopulation compared to all other *emm*89 strains from elsewhere in the UK. For this analysis, we included all *emm*89 genome data reported in the Turner *et al.* manuscript [15] and deposited in publically available databases. Four strains from Scotland were analysed in the Turner *et al.* study, including one each from Dundee, Glasgow, Edinburgh and Kilmarnock (described in Table S1 of the Turner *et al.* manuscript). The great majority of the strains (*n*=122, 93.1 %) were from England localities. Among the 121 clade 3 organisms from our Scotland sources studied herein, several genetically distinct phylogenetic branches were identified (Fig. 1, right). That is, the strains from Scotland sources do not form a single phylogenetic branch sharing a large number of identical polymorphisms relative to a most recent common ancestor. Rather, the clade 3 portion of the rooted-tree has multiple independent branches of short length radiating away from a central hub, suggesting that these strains have evolved fairly randomly away from a relatively recent common ancestor (Fig. 1, right). In combining all 122 strains from Scotland with all 122 strains from England in phylogenetic reconstruction (Fig. 6a), strains were intermixed independent of country on virtually all branches of the clade 3 side of the tree. This is consistent with the Scotland and England strains being a single genetic population. Constraining the reconstruction to only those isolates collected in Scotland (*n*=44) and England (*n*=43) over the same time period 2010–2013 (Fig. 6b), similarly results in the strains being intermixed independent of country of origin.

## Discussion

Our genome sequence data show that with only one exception, all *emm*89 isolates recovered in Scotland since 2010 are members of the recently emerged genetic clade 3. These findings add Scotland to the list of countries in Europe (Finland, Iceland and England) and North America (USA and Canada) where clade 3 *emm*89 strains have been documented by full genome sequencing to be important causes of recent invasive infections and to be genetically related due to descent from a common progenitor.

Clade 3 organisms are noteworthy because they have an evolutionary history involving multiple recent HGT events, which have contributed to their epidemic emergence [6, 15]. Due to one HGT, *emm*89 clade 3 strains lack the *hasABC* gene region required for synthesis of hyaluronic acid capsule, as previously reported for GAS *emm*4 and *emm*22 strains [56]. Capsule was long thought to be a critical *S. pyogenes* virulence factor by virtue of its antiphagocytic properties [57, 58]. Lack of capsule may also affect virulence by altering GAS adherence interactions [59, 60]. Additionally, *emm*89 strains have undergone at least two recent HGT events involving the *nga-ifs-slo* gene region, encoding the secreted toxins NADase and streptolysin O that are documented virulence factors [6, 15]. As a consequence, relative to predecessor clade 1 and clade 2 organisms, clade 3 strains produce an increased amount of these two important toxins [6, 15, 41]. Upregulation of the *nga-ifs-slo* operon and increased production of these two toxins has been shown to enhance virulence of *S. pyogenes* in mouse and non-human primate models of upper respiratory tract infection and severe invasive infection [41, 42]. We recently reported that production of both NADase and SLO are required for full virulence in a mouse infection model [61]. The loss of capsule production and the increased production of NADase and SLO may be closely linked in the emergence of the epidemic clade 3 strains, as it has been shown that when NGA and SLO are highly produced, capsule is no longer required for virulence in a mouse invasive infection model [42].

A further evolution of the *emm*89 clade 3 clone, via an additional HGT event involving the *spyBA* virulence factors [62, 63] characterized an emergent variant, designated subclade 3D, in bacteremic case isolates from Finland [6, 24]. The subclade 3D strains strongly increased in 2014 as a cause of bacteremic infections, and have persisted in Finland. Relative to the five other subclades identified among the bacteremic cases, subclade 3D strains had a significantly higher 30 day case fatality rate. In assessment of phylogeny and recombination among the Scotland *emm89* clade 3 strains, no subclade 3D strains were found, nor were any new HGT events involving other virulence factors. However, the clear propensity for the GAS *emm*89 strains to rapidly evolve through HGT recombinational replacement events generating novel variants of enhanced virulence warrants continued vigilance in monitoring of the epidemic clade 3 clone.

Genomic sequencing has revealed differences in MGE content, phages and ICEs, as the most common source of gene content differences between GAS strains [64]. GAS phages commonly encode one or more secreted virulence factors, such as streptococcal pryogenic exotoxins (*spe* gene superantigens) and streptococcal phage DNases (*spd* gene exonucleases). GAS ICEs commonly encode one or more antibiotic resistance factors, usually for macrolides and tetracyclines. Small and large outbreaks of GAS infections have been associated with the acquisition of phages and/or ICEs. Although no specific ICE was detected in the Scotland cohort, the tetracycline resistance gene for TetM was detected in strain MGAS31641. An association with antibiotic resistance has not been reported to our knowledge in any investigation of the current geographically wide spread increase in *emm*89 infections. All differences in gene content between the Scotland strains were attributable to differences in phage content. Five prophages in nine combinations were found among the 122 Scotland strains (Fig. 2). The phage content, of the 121 Scotland clade 3 strains, mirrored that found for 756 *emm*89 clade 3 strains of the USA, Finland and Iceland [6]. The same three prophage genotypes (PG01, PG02, PG04) in the same ranking were found most prevalent. Moreover, the same *speC* and *spd1* encoding phage was found most prevalent among the Scotland and England *emm*89 clade 3 strains [15]. The paucity of antibiotic resistance and the diversity of prophage profiles found among the *emm*89 clade 3 strains in multiple investigations indicates that no single MGE is conferring a strong selective advantage in the spread and persistence of the epidemic.

Considerable human flux occurs annually across the relatively short (174 km) border between Scotland and England. Thus, we anticipated considerable sharing of common genetic pool of *emm*89 strains between the two countries. To test this idea, we compared the SNP-based phylogeny obtained for this Scotland strain sample with the genome data published previously by Tuner *et al.* for the UK [15] available in public databases. The resultant combined phylogeny demonstrated extensive intermixing of the *emm*89 strains causing infections in these two areas (Fig. 6).

Given the clear recent numerical success of clade 3 organisms in multiple geographically dispersed countries, it is reasonable to speculate that clade 3 strains are present in other countries that have reported an increase in *emm*89 disease activity in recent years. However, full-genome sequence data are required to confirm this suspicion, since neither *emm* gene sequencing nor multi-locus sequence typing provide sufficient genetic information to permit clade identification.

## Supplementary Data

3985 Supplementary File 1

## References

[1] Carapetis JR, Steer AC, Mulholland EK, Weber M (2005). The global burden of group A streptococcal diseases. Lancet Infect Dis.

[2] Smeesters PR, Mcmillan DJ, Sriprakash KS (2010). The streptococcal M protein: a highly versatile molecule. Trends Microbiol.

[3] Sanderson-Smith M, De Oliveira DM, Guglielmini J, McMillan DJ, Vu T (2014). A systematic and functional classification of *Streptococcus pyogenes* that serves as a new tool for molecular typing and vaccine development. J Infect Dis.

[4] Al-Shahib A, Underwood A, Afshar B, Turner CE, Lamagni T (2016). Emergence of a novel lineage containing a prophage in emm/M3 group A *Streptococcus* associated with upsurge in invasive disease in the UK. Microb Genom.

[5] Beres SB, Carroll RK, Shea PR, Sitkiewicz I, Martinez-Gutierrez JC (2010). Molecular complexity of successive bacterial epidemics deconvoluted by comparative pathogenomics. Proc Natl Acad Sci USA.

[6] Beres SB, Kachroo P, Nasser W, Olsen RJ, Zhu L (2016). Transcriptome remodeling contributes to epidemic disease caused by the human pathogen *Streptococcus pyogenes*. MBio.

[7] Davies MR, Holden MT, Coupland P, Chen JH, Venturini C (2015). Emergence of scarlet fever *Streptococcus pyogenes* emm12 clones in Hong Kong is associated with toxin acquisition and multidrug resistance. Nat Genet.

[8] Eraso JM, Olsen RJ, Beres SB, Kachroo P, Porter AR (2016). Genomic landscape of intrahost variation in group A *Streptococcus*: repeated and abundant mutational inactivation of the *fabT* gene encoding a regulator of fatty acid synthesis. Infect Immun.

[9] Fittipaldi N, Beres SB, Olsen RJ, Kapur V, Shea PR (2012). Full-genome dissection of an epidemic of severe invasive disease caused by a hypervirulent, recently emerged clone of group A *Streptococcus*. Am J Pathol.

[10] Fittipaldi N, Olsen RJ, Beres SB, van Beneden C, Musser JM (2012). Genomic analysis of *emm*59 group A *Streptococcus* invasive strains, United States. Emerg Infect Dis.

[11] Nasser W, Beres SB, Olsen RJ, Dean MA, Rice KA (2014). Evolutionary pathway to increased virulence and epidemic group A *Streptococcus* disease derived from 3,615 genome sequences. Proc Natl Acad Sci USA.

[12] Olsen RJ, Fittipaldi N, Kachroo P, Sanson MA, Long SW (2014). Clinical laboratory response to a mock outbreak of invasive bacterial infections: a preparedness study. J Clin Microbiol.

[13] Shea PR, Beres SB, Flores AR, Ewbank AL, Gonzalez-Lugo JH (2011). Distinct signatures of diversifying selection revealed by genome analysis of respiratory tract and invasive bacterial populations. Proc Natl Acad Sci USA.

[14] Teatero S, Coleman BL, Beres SB, Olsen RJ, Kandel C (2017). Rapid emergence of a new clone impacts the population at risk and increases the incidence of type *emm*89 group a streptococcus invasive disease. Open Forum Infect Dis.

[15] Turner CE, Abbott J, Lamagni T, Holden MT, David S (2015). Emergence of a new highly successful acapsular group a *Streptococcus* clade of genotype *emm8*9 in the United Kingdom. MBio.

[16] Creti R, Imperi M, Baldassarri L, Pataracchia M, Recchia S (2007). emm Types, virulence factors, and antibiotic resistance of invasive *Streptococcus pyogenes* isolates from Italy: What has changed in 11 years?. J Clin Microbiol.

[17] Darenberg J, Luca-Harari B, Jasir A, Sandgren A, Pettersson H (2007). Molecular and clinical characteristics of invasive group A streptococcal infection in Sweden. Clin Infect Dis.

[18] Falkenhorst G, Bagdonaite J, Lisby M, Madsen SB, Lambertsen L (2008). Outbreak of group A streptococcal throat infection: don't forget to ask about food. Epidemiol Infect.

[19] Friães A, Machado MP, Pato C, Carriço J, Melo-Cristino J (2015). Emergence of the same successful clade among distinct populations of *emm*89 *Streptococcus pyogenes* in multiple geographic regions. MBio.

[20] Friães A, Pinto FR, Silva-Costa C, Ramirez M, Melo-Cristino J (2012). Group A streptococci clones associated with invasive infections and pharyngitis in Portugal present differences in emm types, superantigen gene content and antimicrobial resistance. BMC Microbiol.

[21] Ikebe T, Tominaga K, Shima T, Okuno R, Kubota H (2015). Increased prevalence of group A streptococcus isolates in streptococcal toxic shock syndrome cases in Japan from 2010 to 2012. Epidemiol Infect.

[22] Karaky NM, Araj GF, Tokajian ST (2014). Molecular characterization of *Streptococcus pyogenes* group A isolates from a tertiary hospital in Lebanon. J Med Microbiol.

[23] Koh E, Kim S (2010). Decline in erythromycin resistance in group A Streptococci from acute pharyngitis due to changes in the emm Genotypes rather than restriction of antibiotic use. Korean J Lab Med.

[24] Latronico F, Nasser W, Puhakainen K, Ollgren J, Hyyryläinen HL (2016). Genomic characteristics behind the spread of bacteremic group A *Streptococcus* type *emm*89 in Finland, 2004-2014. J Infect Dis.

[25] Lepoutre A, Doloy A, Bidet P, Leblond A, Perrocheau A (2011). Epidemiology of invasive *Streptococcus* pyogenes infections in France in 2007. J Clin Microbiol.

[26] Luca-Harari B, Darenberg J, Neal S, Siljander T, Strakova L (2009). Clinical and microbiological characteristics of severe *Streptococcus pyogenes* disease in Europe. J Clin Microbiol.

[27] Naseer U, Steinbakk M, Blystad H, Caugant DA (2016). Epidemiology of invasive group A streptococcal infections in Norway 2010-2014: a retrospective cohort study. Eur J Clin Microbiol Infect Dis.

[28] Nelson GE, Pondo T, Toews KA, Farley MM, Lindegren ML (2016). Epidemiology of invasive group A Streptococcal infections in the United States, 2005-2012. Clin Infect Dis.

[29] O'Loughlin RE, Roberson A, Cieslak PR, Lynfield R, Gershman K (2007). The epidemiology of invasive group A streptococcal infection and potential vaccine implications: United States, 2000-2004. Clin Infect Dis.

[30] Olafsdottir LB, Erlendsdóttir H, Melo-Cristino J, Weinberger DM, Ramirez M (2014). Invasive infections due to Streptococcus pyogenes: seasonal variation of severity and clinical characteristics, Iceland, 1975 to 2012. Euro Surveill.

[31] Plainvert C, Doloy A, Loubinoux J, Lepoutre A, Collobert G (2012). Invasive group A streptococcal infections in adults, France (2006-2010). Clin Microbiol Infect.

[32] Plainvert C, Loubinoux J, Bidet P, Doloy A, Touak G (2014). [Epidemiology of *Streptococcus pyogenes* invasive diseases in France (2007–2011)]. Arch Pediatr.

[33] Rantala S, Vähäkuopus S, Siljander T, Vuopio J, Huhtala H (2012). *Streptococcus pyogenes* bacteraemia, emm types and superantigen profiles. Eur J Clin Microbiol Infect Dis.

[34] Shea PR, Ewbank AL, Gonzalez-Lugo JH, Martagon-Rosado AJ, Martinez-Gutierrez JC (2011). Group A *Streptococcus emm* gene types in pharyngeal isolates, Ontario, Canada, 2002**–**2010. Emerg Infect Dis.

[35] Siljander T, Lyytikäinen O, Vähäkuopus S, Snellman M, Jalava J (2010). Epidemiology, outcome and emm types of invasive group A streptococcal infections in Finland. Eur J Clin Microbiol Infect Dis.

[36] Smit PW, Lindholm L, Lyytikäinen O, Jalava J, Pätäri-Sampo A (2015). Epidemiology and emm types of invasive group A streptococcal infections in Finland, 2008-2013. Eur J Clin Microbiol Infect Dis.

[37] Tamayo E, Montes M, García-Arenzana JM, Pérez-Trallero E (2014). *Streptococcus pyogenes emm*-types in northern Spain; population dynamics over a 7-year period. J Infect.

[38] Vähäkuopus S, Vuento R, Siljander T, Syrjänen J, Vuopio J (2012). Distribution of *emm* types in invasive and non-invasive group A and G streptococci. Eur J Clin Microbiol Infect Dis.

[39] Williamson DA, Moreland NJ, Carter P, Upton A, Morgan J (2014). Molecular epidemiology of group A streptococcus from pharyngeal isolates in Auckland, New Zealand, 2013. N Z Med J.

[40] Williamson DA, Morgan J, Hope V, Fraser JD, Moreland NJ (2015). Increasing incidence of invasive group A streptococcus disease in New Zealand, 2002-2012: a national population-based study. J Infect.

[41] Zhu L, Olsen RJ, Nasser W, Beres SB, Vuopio J (2015). A molecular trigger for intercontinental epidemics of group A Streptococcus. J Clin Invest.

[42] Zhu L, Olsen RJ, Nasser W, de La Riva Morales I, Musser JM (2015). Trading capsule for increased cytotoxin production: contribution to virulence of a newly emerged clade of emm89 *Streptococcus pyogenes*. MBio.

[43] Liu YM, Zhao JZ, Li BB, Yang JY, Dong XG (2014). A report on the first outbreak of a single clone group A Streptococcus (emm-type 89) tonsillopharyngitis in China. J Microbiol Immunol Infect.

[44] Lindsay DS, Brown AW, Scott KJ, Denham B, Thom L (2016). Circulating emm types of *Streptococcus pyogenes* in Scotland: 2011-2015. J Med Microbiol.

[45] Bolger AM, Lohse M, Usadel B (2014). Trimmomatic: a flexible trimmer for Illumina sequence data. Bioinformatics.

[46] Liu Y, Schröder J, Schmidt B (2013). Musket: a multistage k-mer spectrum-based error corrector for Illumina sequence data. Bioinformatics.

[47] Bankevich A, Nurk S, Antipov D, Gurevich AA, Dvorkin M (2012). SPAdes: a new genome assembly algorithm and its applications to single-cell sequencing. J Comput Biol.

[48] Inouye M, Dashnow H, Raven LA, Schultz MB, Pope BJ (2014). SRST2: Rapid genomic surveillance for public health and hospital microbiology labs. Genome Med.

[49] Garrison E, Marth (2012). Haplotype-based variant detection from short-read sequencing. arXiv:1207.3907 [q-bio.GN].

[50] Croucher NJ, Page AJ, Connor TR, Delaney AJ, Keane JA (2015). Rapid phylogenetic analysis of large samples of recombinant bacterial whole genome sequences using gubbins. Nucleic Acids Res.

[51] Huson DH, Bryant D (2006). Application of phylogenetic networks in evolutionary studies. Mol Biol Evol.

[52] Huson DH, Scornavacca C (2012). Dendroscope 3: an interactive tool for rooted phylogenetic trees and networks. Syst Biol.

[53] Kumar S, Stecher G, Tamura K (2016). MEGA7: molecular evolutionary genetics analysis version 7.0 for bigger datasets. Mol Biol Evol.

[54] Rambaut A, Lam TT, Max Carvalho L, Pybus OG (2016). Exploring the temporal structure of heterochronous sequences using TempEst (formerly Path-O-Gen). Virus Evol.

[55] Quinlan AR, Hall IM (2010). BEDTools: a flexible suite of utilities for comparing genomic features. Bioinformatics.

[56] Flores AR, Jewell BE, Fittipaldi N, Beres SB, Musser JM (2012). Human disease isolates of serotype m4 and m22 group a streptococcus lack genes required for hyaluronic acid capsule biosynthesis. MBio.

[57] Foley MJ, Wood WB (1959). Studies on the pathogenicity of group A streptococci. II. The antiphagocytic effects of the M protein and the capsular gel. J Exp Med.

[58] Wessels MR, Moses AE, Goldberg JB, Dicesare TJ (1991). Hyaluronic acid capsule is a virulence factor for mucoid group A streptococci. Proc Natl Acad Sci USA.

[59] Darmstadt GL, Mentele L, Podbielski A, Rubens CE (2000). Role of group A streptococcal virulence factors in adherence to keratinocytes. Infect Immun.

[60] Whitnack E, Bisno AL, Beachey EH (1981). Hyaluronate capsule prevents attachment of group A streptococci to mouse peritoneal macrophages. Infect Immun.

[61] Zhu L, Olsen RJ, Lee JD, Porter AR, Deleo FR (2017). Contribution of secreted nadase and streptolysin O to the pathogenesis of epidemic serotype M1 *Streptococcus pyogenes* infections. Am J Pathol.

[62] Edgar RJ, Chen J, Kant S, Rechkina E, Rush JS (2016). SpyB, a small heme-binding protein, affects the composition of the cell wall in *Streptococcus pyogenes*. Front Cell Infect Microbiol.

[63] Hoff JS, Dewald M, Moseley SL, Collins CM, Voyich JM (2011). SpyA, a C3-like ADP-ribosyltransferase, contributes to virulence in a mouse subcutaneous model of Streptococcus pyogenes infection. Infect Immun.

[64] Beres SB, Musser JM (2007). Contribution of exogenous genetic elements to the group A *Streptococcus* metagenome. PLoS One.

