# Supplementary material for: Genome sequence analysis of emm89 Streptococcus pyogenes strains causing infections in Scotland, 2010–2016
Source: J Med Microbiol. 2017 Nov 3;66(12):1765–73. doi: 10.1099/jmm.0.000622 (PMC5845742; doi:10.1099/jmm.0.000622)
Supplement: Supplementary File 1 [file jmm-66-1765-s001.pdf]

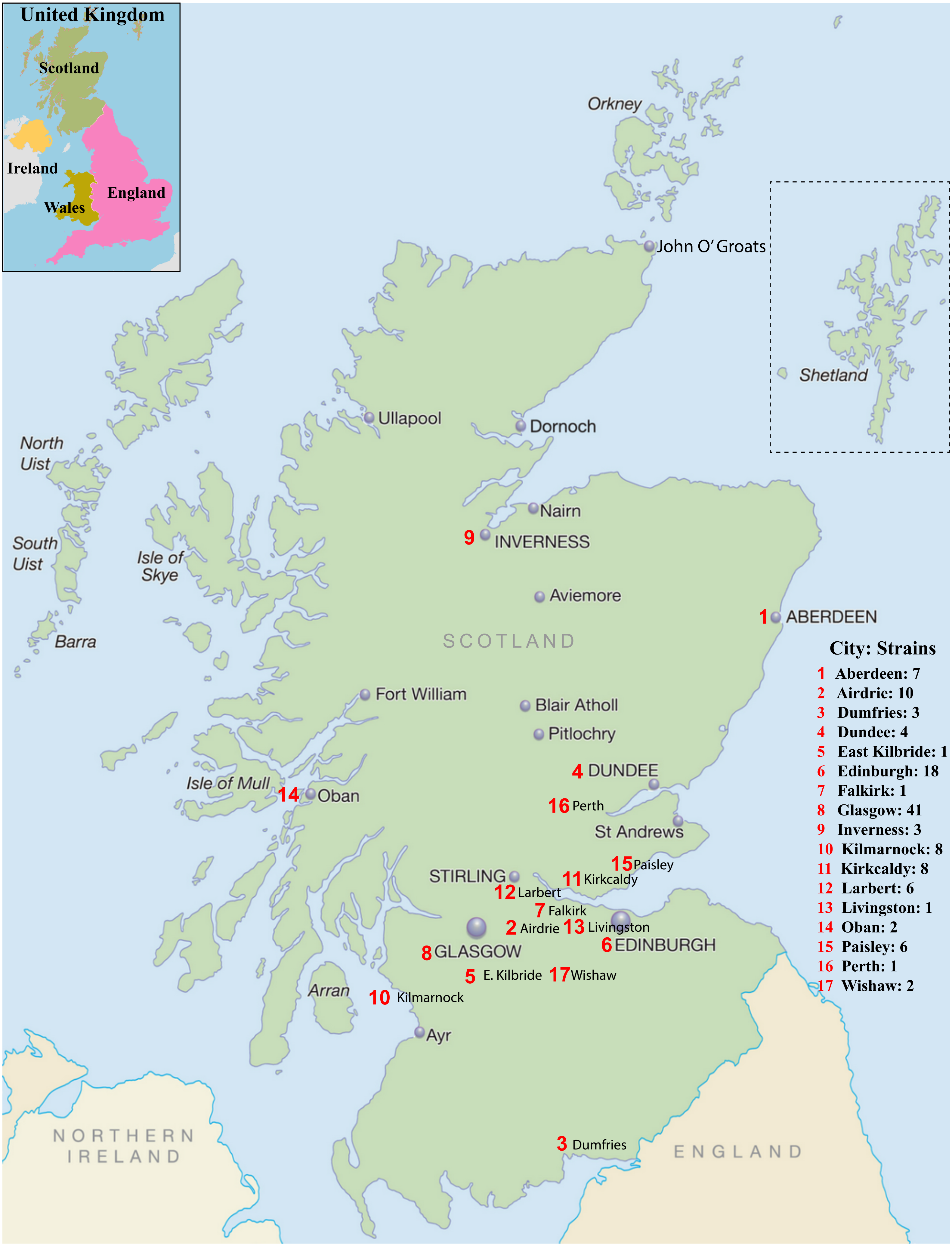
United Kingdom
Scotland
Ireland
England
Wales
Orkney
John O' Groats
Shetland
Ullapool
Dornoch
North Uist
South Uist
Isle of Skye
Barra
Nairn
9 INVERNESS
Aviemore
1 ABERDEEN
SCOTLAND
Fort William
Blair Atholl
Pitlochry
4 DUNDEE
Isle of Mull
14 Oban
16 Perth
St Andrews
15 Paisley
STIRLING
11 Kirkcaldy
12 Larbert
7 Falkirk
2 Airdrie
13 Livingston
6 EDINBURGH
8 GLASGOW
5 E. Kilbride
17 Wishaw
Arran
10 Kilmarnock
Ayr
NORTHERN IRELAND
3 Dumfries
ENGLAND
City: Strains
1 Aberdeen: 7
2 Airdrie: 10
3 Dumfries: 3
4 Dundee: 4
5 East Kilbride: 1
6 Edinburgh: 18
7 Falkirk: 1
8 Glasgow: 41
9 Inverness: 3
10 Kilmarnock: 8
11 Kirkcaldy: 8
12 Larbert: 6
13 Livingston: 1
14 Oban: 2
15 Paisley: 6
16 Perth: 1
17 Wishaw: 2

Table S1. Scotland strain characteristics.

No.	Strain	Year	Infection Type	Geographic Location	Prophage Profile*
1	MGAS31642	2010	Invasive	Glasgow	1
2	MGAS31643	2010	Invasive	Glasgow	2
3	MGAS31646	2010	Invasive	Glasgow	2
4	MGAS31647	2010	Invasive	Glasgow	2
5	MGAS31648	2010	Invasive	Glasgow	2
6	MGAS31649	2010	Invasive	Glasgow	2
7	MGAS31683	2010	Invasive	Dundee	1
8	MGAS31684	2010	Invasive	Edinburgh	6
9	MGAS31686	2010	Invasive	Aberdeen	3
10	MGAS31845	2010	Invasive	Glasgow	2
11	MGAS31847	2010	Invasive	Glasgow	2
12	MGAS31848	2010	Invasive	Glasgow	2
13	MGAS31621	2011	Invasive	Airdrie	1
14	MGAS31623	2011	Invasive	Glasgow	1
15	MGAS31627	2011	Noninvasive	Glasgow	1
16	MGAS31628	2011	Noninvasive	Glasgow	1
17	MGAS31629	2011	Noninvasive	Glasgow	1
18	MGAS31630	2011	Noninvasive	Glasgow	1
19	MGAS31632	2011	Noninvasive	Glasgow	1
20	MGAS31633	2011	Invasive	Glasgow	1
21	MGAS31634	2011	Noninvasive	Glasgow	1
22	MGAS31636	2011	Invasive	Wishaw	1
23	MGAS31638	2011	Invasive	Glasgow	1
24	MGAS31639	2011	Invasive	Glasgow	1
25	MGAS31640	2011	Invasive	Glasgow	1
26	MGAS31641	2011	Invasive	Glasgow	1
27	MGAS31644	2011	Invasive	Glasgow	3

28	MGAS31645	2011	Invasive	Glasgow	2
29	MGAS31650	2011	Invasive	Glasgow	1
30	MGAS31651	2011	Noninvasive	Glasgow	1
31	MGAS31661	2011	Invasive	Airdrie	6
32	MGAS31690	2011	Invasive	Kilmarnock	1
33	MGAS31652	2012	Noninvasive	Airdrie	2
34	MGAS31653	2012	Noninvasive	Airdrie	2
35	MGAS31654	2012	Noninvasive	Airdrie	2
36	MGAS31655	2012	Noninvasive	Airdrie	2
37	MGAS31656	2012	Noninvasive	Glasgow	1
38	MGAS31657	2012	Noninvasive	Glasgow	1
39	MGAS31659	2012	Noninvasive	Glasgow	1
40	MGAS31660	2012	Noninvasive	Glasgow	1
41	MGAS31672	2012	Invasive	Aberdeen	1
42	MGAS31682	2012	Noninvasive	Glasgow	2
43	MGAS31693	2012	Invasive	Kilmarnock	1
44	MGAS31673	2013	Invasive	Kilmarnock	4
45	MGAS31674	2013	Invasive	Inverness	3
46	MGAS31676	2013	Invasive	Edinburgh	1
47	MGAS31714	2013	Invasive	Perth	3
48	MGAS31716	2013	Invasive	Kilmarnock	1
49	MGAS31717	2013	Invasive	Kirkcaldy	3
50	MGAS31718	2013	Invasive	Dumfries	1
51	MGAS31677	2014	Noninvasive	Edinburgh	1
52	MGAS31678	2014	Noninvasive	Edinburgh	1
53	MGAS31679	2014	Invasive	Glasgow	2
54	MGAS31680	2014	Invasive	Glasgow	1
55	MGAS31687	2014	Invasive	Edinburgh	1
56	MGAS31688	2014	Invasive	East Kilbride	2

57	MGAS31713	2014	Invasive	Glasgow	2
58	MGAS31720	2014	Invasive	Paisley	1
59	MGAS31722	2014	Noninvasive	Edinburgh	1
60	MGAS31723	2014	Invasive	Kirkcaldy	3
61	MGAS31725	2014	Invasive	Glasgow	6
62	MGAS31726	2014	Invasive	Airdrie	1
63	MGAS31727	2014	Invasive	Inverness	1
64	MGAS31729	2014	Invasive	Paisley	2
65	MGAS31731	2014	Invasive	Glasgow	2
66	MGAS31732	2014	Invasive	Wishaw	1
67	MGAS31733	2014	Invasive	Larbert	8
68	MGAS31734	2014	Invasive	Glasgow	2
69	MGAS31735	2014	Invasive	Glasgow	1
70	MGAS31849	2014	Invasive	Kilmarnock	1
71	MGAS31850	2014	Invasive	Airdrie	1
72	MGAS31851	2014	Invasive	Paisley	3
73	MGAS31852	2014	Invasive	Kilmarnock	1
74	MGAS31853	2014	Invasive	Airdrie	1
75	MGAS31854	2014	Invasive	Inverness	2
76	MGAS31855	2014	Invasive	Kirkcaldy	3
77	MGAS31856	2014	Invasive	Paisley	5
78	MGAS31857	2014	Invasive	Aberdeen	2
79	MGAS31858	2014	Invasive	Paisley	2
80	MGAS31859	2014	Invasive	Edinburgh	1
81	MGAS31662	2015	Noninvasive	Aberdeen	1
82	MGAS31670	2015	Invasive	Dumfries	1
83	MGAS31681	2015	Invasive	Edinburgh	2
84	MGAS31737	2015	Invasive	Edinburgh	2
85	MGAS31738	2015	Invasive	Larbert	4

86	MGAS31739	2015	Invasive	Dundee	2
87	MGAS31740	2015	Invasive	Edinburgh	1
88	MGAS31741	2015	Invasive	Oban	5
89	MGAS31742	2015	Invasive	Larbert	1
90	MGAS31743	2015	Invasive	Larbert	1
91	MGAS31744	2015	Invasive	Glasgow	2
92	MGAS31745	2015	Invasive	Livingston	2
93	MGAS31746	2015	Invasive	Larbert	1
94	MGAS31747	2015	Invasive	Airdrie	1
95	MGAS31748	2015	Invasive	Falkirk	2
96	MGAS31749	2015	Invasive	Edinburgh	1
97	MGAS31751	2015	Invasive	Edinburgh	3
98	MGAS31753	2015	Noninvasive	Aberdeen	1
99	MGAS31861	2015	Invasive	Aberdeen	2
100	MGAS31862	2015	Invasive	Edinburgh	2
101	MGAS31863	2015	Invasive	Edinburgh	3
102	MGAS31864	2015	Invasive	Dundee	3
103	MGAS31865	2015	Invasive	Dundee	3
104	MGAS31866	2015	Invasive	Edinburgh	1
105	MGAS31867	2015	Invasive	Kirkcaldy	1
106	MGAS31868	2015	Invasive	Edinburgh	3
107	MGAS31869	2015	Invasive	Larbert	1
108	MGAS31871	2015	Invasive	Aberdeen	2
109	MGAS31872	2015	Invasive	Kirkcaldy	1
110	MGAS31873	2015	Invasive	Kilmarnock	4
111	MGAS31663	2016	Invasive	Oban	5
112	MGAS31664	2016	Invasive	Kilmarnock	7
113	MGAS31665	2016	Invasive	Kirkcaldy	2
114	MGAS31666	2016	Invasive	Kirkcaldy	4

115	MGAS31667	2016	Noninvasive	Edinburgh	2
116	MGAS31669	2016	Invasive	Glasgow	2
117	MGAS31671	2016	Invasive	Dumfries	1
118	MGAS31675	2016	Invasive	Glasgow	9
119	MGAS31689	2016	Invasive	Edinburgh	1
120	MGAS31691	2016	Noninvasive	Kirkcaldy	2
121	MGAS31692	2016	Invasive	Paisley	1
122	MGAS31712	2016	Noninvasive	Glasgow	1

* Prophage profiles are as defined in Fig. 2A.
